# Nutritional Quality of School Meals in France: Impact of Guidelines and the Role of Protein Dishes

**DOI:** 10.3390/nu10020205

**Published:** 2018-02-13

**Authors:** Florent Vieux, Christophe Dubois, Christelle Duchêne, Nicole Darmon

**Affiliations:** 1 MS-Nutrition, 13385 Marseille CEDEX 05, France; florent.vieux@ms-nutrition.com; 2 Trophis, 13170 Les Pennes Mirabeau, France; solce@free.fr; 3 CIV–Viande, Sciences et Société, 207, rue de Bercy, TSA 41309, 75564 Paris CEDEX 12, France; c.duchene@interbev.fr; 4 UMR NORT (Mixed Research Unit—Nutrition, Obesity and Risk of Thrombosis), INRA 1260, Aix-Marseille University, INSERM, 13005 Marseille, France; 5 MOISA, INRA, CIHEAM-IAMM, CIRAD, Montpellier SupAgro, Université de Montpellier, 34060 Montpellier CEDEX 2, France

**Keywords:** schools, diet, children, France, policy, meat, protein, nutrition, sustainability

## Abstract

In France, school meals must comply with 15 frequency criteria (FC) expressed as nutritional requirements (e.g., “starters containing more than 15% fat served no more than four times out of 20”) in a series of 20 consecutive meals. The objective was to assess, for the first time, the nutritional impact of complying with French school food FC. Based on 40 series of meals actually served in primary schools (“observed series”), several scenarios (1600 series per scenario) of compliance or non-compliance with FC were simulated, and nutritional quality was assessed via the mean adequacy ratio (MAR/2000 kcal). In the observed series, only 9.7 FC on average (range 4–14) were fulfilled. In the simulated series: (i) MAR was positively associated with FC compliance level, with the highest MAR obtained with complete compliance; (ii) MAR decreased when meat or fish-based dishes were replaced by dishes without meat or fish; and (iii) removing the protein dish without replacement led to the lowest MAR. This study demonstrates that French school food guidelines ensure good nutritional quality of food services. It also shows that generalizing the service of meals without meat or fish would deteriorate overall nutritional quality, highlighting the need to define the composition of vegetarian dishes and their frequency of service to children.

## 1. Introduction

The European Union Action Plan on childhood obesity [1] and many other official documents—listed in a recent report by the European Commission [2]—underscore the fact that schools provide a privileged framework to promote healthy behaviors, notably in regard to physical activity and food. This report [2] states that the provision of healthy balanced meals in schools can improve dietary habits, lessen the incidence of childhood overweight and obesity, increase school attendance and performance, help reduce health inequalities, and raise children’s and school’s health awareness. All the member States have made considerable progress in this field with the establishment of either voluntary or mandatory school food policies [3]. However, the practical implementation of these policies in terms of food offered in school settings remains complex [2].

The World Health Organization (WHO) regional office [4] and the European Parliament [5] have both emphasized the need to offer children healthier food at school, notably by developing or improving nutritional guidelines for school meals. School meals often have better nutritional quality than meals eaten outside the school [6,7]. Most European countries have established specific recommendations for school meals, but only the United Kingdom, Portugal [8], and more recently France [9,10] have made them mandatory.

In France, the entire public catering sector has been the object of nutritional guidelines since 2001. These guidelines were elaborated and are regularly updated [11,12,13] by the Groupe d’Etude des Marchés Restauration Collective et Nutrition (GEM-RCN (public catering and nutrition market study group)), a consultation body that includes the main public and private partners involved in public catering in France, officially brought together under the auspices of the French Ministry of the Economy. These guidelines pursue a nutritional and public health goal while taking into account the constraints and practices of professionals in the field, diners’ food habits, and the desire to foster conviviality and educate children about dietary diversity. The GEM-RCN’s nutrition guidelines define the general format of meals, set portion sizes for the dishes and foods most frequently served in public catering, and recommend service frequencies for fifteen types of dishes in a series of 20 consecutive lunches (or approximately four weeks of school). These 15 frequency criteria became mandatory in all schools in 2011 [9,10]. The studies conducted by the Agence Nationale de Sécurité Sanitaire de l’Alimentation, de l’Environnement et du Travail (ANSES (the French agency for food, environmental and occupational health safety)) before the guidelines became mandatory had shown that these criteria were not known widely enough, explaining insufficient application by professionals [14,15]. In addition, quantification of the nutritional impact of these guidelines remains limited [15], which may weaken application.

In addition to nutritional goals, school food is also subject to other expectations such as taste education, respect for food traditions and habits, support of local agriculture, the fight against waste, and environmental protection [16]. Protein dishes in particular are the subject of multiple considerations. The meat/egg/fish food group is a privileged source of many essential nutrients in the French diet, and the French spend more than one quarter of their food budgets on it [17]. These nutritional and economic specificities—which prevail in the same manner for protein dishes in public catering—are complicated by environmental concerns [18,19]. Indeed, according to a report by the Agence de l’Environnement et de la Maîtrise de l’Énergie (ADEME (environment and energy control agency)), meat and fish make up 21% of food loss and waste in public catering (schools, healthcare and companies) in France, while accounting for 46% of costs and 77% of greenhouse gas emissions generated by these losses [20]. It is therefore important to look at replacement protein sources for sustainability reasons [19]. Protein dishes also raise sociocultural issues because meat and, to a lesser extent, fish are the subject of specific requests (e.g., exclusion from meals) by individuals and population groups that emphasize certain religious, ethic, political, psychological and ideological values, reasons and principles [21].

Based on meals actually served in primary schools, the objective of the present study was to assess, for the first time, the nutritional impact of complying with French school food guidelines, and of non compliance scenarios, including modifications in protein dishes.

## 2. Materials and Methods

**Figure 1:**
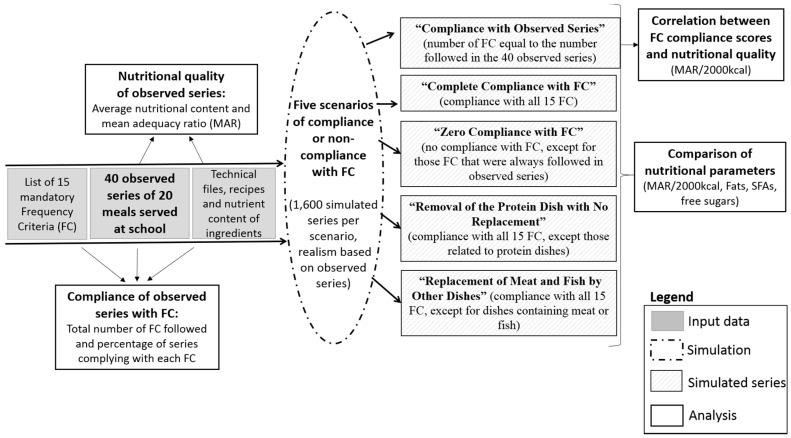
Overview of the methodology used to generate simulated series of 20 consecutive meals (based on 40 observed series), according to five scenarios of compliance and non-compliance with the 15 frequency criteria (FC) in French school food guidelines.

The GEM-RCN’s nutrition guidelines [13] for public catering define the general format of meals (four or five components: Starter, protein dish, side dish, dairy product, and dessert), the recommended portion sizes for the dishes and foods most frequently served in public catering, and the recommended service frequencies for 15 types of dishes in a series of 20 consecutive lunches (or approximately 4 weeks of school). These 15 types of dishes are themselves defined by criteria based on one or more characteristic(s) such as the meal component (starter, for example), and/or the content of some food groups in the dish (for example, proportion of vegetables), and/or certain nutrients (fat content, for example), or other characteristics of the food served (raw/cooked, ground/not ground, etc.). The combination of a criterion and an associated frequency forms a “frequency criterion”. For example, dishes meeting the criterion “starter containing more than 15% fat” must be served no more than four times in a series of 20 consecutive meals. Table 1 lists the 15 frequency criteria (referred to as “FC” hereafter). Compliance with these 15 FC became mandatory for all schools in 2011 [9,10], while portion sizes remain indicative. Figure 1 provides a general overview of the methodology. Input data were series of meals actually served in primary schools (“observed series”), the list of the 15 mandatory FC, technical files, recipes and nutrient content of ingredients. To assess the nutritional impact of French school food guidelines, series of meals were simulated according to five scenarios of compliance or non-compliance with FC, including modifications regarding the protein dish.

### 2.1. Data Collection

For this study, data were collected on 40 series of 20 lunches actually served in primary schools in France. These series are referred to as “observed series” hereafter. They were provided by two partners in the study: The Syndicat National de la Restauration Collective (SNRC (national public catering union)) to whom the production and management of school meals has been delegated; and Restau’Co, a network of directly managed public catering professionals. The two partners wrote to their networks with the operational aim of securing the participation in the study of approximately ten organizations each, seeking the widest possible diversity in terms of type of operation (meals produced on site or in a central kitchen), number of employees, geographic location, city size, and number of meals served. For each organization, participation consisted of providing two series, one during spring (mainly May and June) and the other during fall (mainly October and November). Forty observed series were thus collected (2 partners × 10 organizations from each partner × 2 series from each organization). For each series of 20 meals, the names of the dishes were listed, and a technical file—i.e., the ingredients and quantities used—was provided for each dish. Technical files were also provided for simple foods such as a banana or yoghurt. Specifications were sent to each organization to ensure maximum data homogeneity, quality and precision. Each series was composed of 20 lunches served over a period of 20 consecutive school days. The near totality of meals (94%) consisted of 5 components: Starter, protein dish, side dish, dairy product and dessert.

The ingredients making up each dish were recorded by an expert in the field (a dietician) and the nutrient composition of each dish was estimated using the French food nutrient composition reference table [22]. Each dish was identified according to its meal component (for example, starter) and the food category of its main ingredient (e.g., vegetables) (Table A1). Then, a classification specific to the 15 FC (Table 1) was applied (for example, the “starter containing more than 15% fat” criterion).

A portion of 50 g of bread was added to each meal as the two partners involved in the study indicated that this is the portion of bread usually offered with lunch at school.

For each dish we estimated two weights:

- The “observed” weight was determined by adding the weight of each ingredient indicated on the technical file (taking into account standard cooking loss factors) divided by the number of diners;
- the “recommended” weight was derived from the weights indicated for the various types of dishes in the GEM-RCN guidelines [13].

As explained below, observed weights were applied to the observed series and recommended weights were used for the simulated series.

### 2.2. Nutritional Quality Indicators

The nutritional quality of each 20-meal series was estimated through the Mean Adequacy Ratio (MAR), an indicator that estimates the mean content of essential nutrients expressed as a percentage of recommended intakes [23]. In the present study, the MAR was calculated taking into account 23 nutrients (proteins, fiber, essential fatty acids, vitamins and minerals) as indicated in Equation (1).

(1) $$\mathrm{MAR}=\frac{1}{23}\times \sum_{n=1}^{23}\frac{content_{n}}{reco_{n}\times 20}\times 100$$

where *content_n_* is the total amount of *nutrient_n_* in the series of meals considered and *reco_n_* is the recommended daily intake of this same nutrient. The recommended daily intake was multiplied by 20 to take into account the number of meals in the series.

The recommended daily intakes applied in the present study for each of these nutrients are given in Table 2. These values were obtained by weighting the recommended dietary allowance in France [24] according to the age and gender representativeness of the population concerned, specifically children aged 4 to 13 years attending primary school [25]. For example, the calcium recommendation in the present study (924 mg) was obtained by considering the age-specific recommendations (i.e., 700 mg for 4- to 6-year-olds, 900 mg for 7- to 9-year-olds and 1200 mg for 10- to 13-year-olds, irrespective of gender) and the representativeness of each age bracket (19.8%, 58.9% and 21.3% respectively). The MAR was calculated for each series of 20 meals since the series is the relevant unit in the guidelines. The MAR was also expressed for 2000 kcal to have a view of the nutritional quality of the series assuming it was the sole dietary source for a benchmark daily energy need of 2000 kcal. In this second version of the MAR, each *content_n_* was calculated for 2000 kcal and *reco_n_* was therefore not multiplied by 20, as indicated in Equation (2).

(2) $$\mathrm{MAR}/2000\;\mathrm{kcal}=\frac{1}{23}\times \sum_{n=1}^{23}\frac{\frac{content_{n}}{nrj}\times 2000}{reco_{n}}\times 100,$$

where *content_n_* is the total amount of nutrient *n* in the series of meals considered, *nrj* is the energy content of the series, and *reco_n_* is the recommended daily intake of nutrient *n*.

In both MAR and MAR/2000 kcal, the ratio for each nutrient was truncated at 100 so that a high content of one nutrient could not compensate for the low intake of others [23]. Thus, the MAR and MAR per 2000 kcal had a maximum theoretical score of 100. An example of calculation of MAR and MAR/2000 kcal for one given series is provided in Table A2.

The amounts of fats, saturated fatty acids and free sugars were also calculated. Free sugars are defined by the WHO as sugar added to foods plus sugars naturally present in honey, syrups and fruit juices [26].

### 2.3. FC Compliance Scores

Two scores were developed to estimate the level of compliance with FC. The “Number of FC Followed” score is the sum of 15 sub-scores corresponding to compliance with each of the 15 FC. If the series complied with one criterion, a sub-score of 1 was assigned; if it did not comply, the sub-score was 0 Series could therefore obtain a score ranging from 0 to 15. However, when the series did not comply with a frequency criterion, this score had the inconvenience of not taking into account distance from expected frequency. For this reason, another score, called the “Relative Compliance Score” was developed. This score also ranges from 0 to 15 and is the sum of 15 sub-scores corresponding to compliance with each of the 15 FC. However, when a criterion was not met, a sub-score of less than 1, but as close to 1 as the observed frequency was close to expected frequency, was assigned. In this case, the sub-score was equal to (1 minus the absolute value of the relative gap), with the relative gap equaling ((observed frequency minus recommended frequency)/recommended frequency).

For example, for the “starter containing more than 15% fat: 4/20 max” criterion, a series containing four starters of this type received a sub-score of 1 for each of the two scores; a series that contained 5 starters of this type received a sub-score of 0 for the first score and a sub-score of 0.75 for the second score; and a series that contained 7 starters of this type obtained a sub-score of 0 for the first score and a sub-score of 0.25 for the second score.

### 2.4. Generating Simulated Series

Simulations were performed to analyze the relationship between compliance with FC and nutritional quality. This was done because of the anticipated absence, in the observed sample, of series complying with all 15 FC and of series not complying at all, as well as of series without meat or fish. Five different situations were simulated using the five scenarios (Figure 1).

For each scenario, 1600 series of meals were generated, corresponding to 32,000 meals in each scenario, or 160,000 meals in all. For all the scenarios, only recommended amounts were applied to the weight of each dish in the simulations in order to study the nutritional impact of FC specifically (i.e., without the portion sizes actually served in the observed series interfering with the results). Methodological details describing each of the five scenarios are provided in Appendix A.

### 2.5. Statistical Analysis

The distribution of the number of FC followed in the 40 observed series was calculated, as well as the percentage of series complying with each criterion. Nutritional values were calculated for each 20-meal series because the series is the relevant unit in the guidelines. Some results were presented after being divided by 20, to obtain a “per meal” figure that can be interpreted more directly.

For macronutrients, the values were expressed as a percentage of energy content. For fiber, vitamins and minerals, the values were expressed as a percentage of recommended daily nutrient intakes and compared to the percentage of recommended daily energy intake provided by the series, assuming that good nutritional density would be achieved when the percentage of recommended daily nutrient intakes was higher than the percentage of recommended daily energy. This was done by analogy with the concept of nutritious food defined as foods that provide proportionally more nutrients to favor than calories [27]. In scenario 1 (“Compliance with Observed Series”), the association between FC compliance scores and nutritional quality (estimated by MAR/2000 kcal) was analyzed, represented by point clouds and tested by Spearman correlation coefficients. Finally, the averages for MAR/2000 kcal, fat content, saturated fatty acid content, and free sugar content were compared across the five scenarios using Bonferroni-adjusted General Linear Model (GLM) statistical analysis. The variations in these indicators across the different scenarios were also compared to verify the spread difference of certain distributions.

## 3. Results

### 3.1. Distribution of Dishes in the Observed Series

The starters were above all composed of vegetables; protein dishes were composed mainly of pork, poultry, beef, veal, lamb or fish; dairy products were mostly cheese; side dishes contained starches and vegetables; and desserts were mainly fruit, and less often sugar products and dairy desserts (Table A1).

### 3.2. FC Compliance in Observed Series

**Figure 2:**
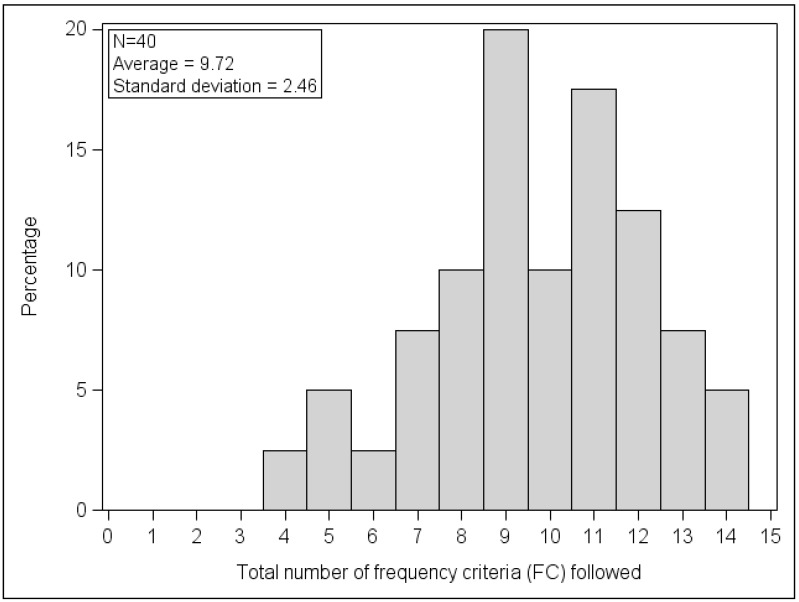
Distribution of the number of frequency criteria complied with in the observed series of meals (*N* = 40 series).

On average, 9.7 out of the 15 FC were fulfilled in the sample of 40 observed series, with a minimum of 4 and a maximum of 14 (Figure 2).

The percentage of series fulfilling the frequency criteria in the 40 observed series varied depending on the criterion considered (Table 1).

Only two individual FC were systematically followed in all 40 series: “dishes to fry or pre-fried dishes containing more than 15% fat” (4/20 max) and “desserts or dairy products containing more than a total of 20 g of simple sugars per portion and less than 15% fat” (4/20 max). The FC on “starters containing more than 15% fat” (4/20 max) and “desserts containing more than 15% fat” (3/20 max) were also among those with the highest percentage of fulfillment. Criteria expressed by strict equality—“cooked vegetables other than pulses, alone or in a mixture containing at least 50% vegetables” (=10/20) and “pulses, starches or grains, alone or in a mixture containing at least 50% of pulses, starches or grains” (=10/20)—showed low percentage of fulfillment (27.5% each). The criterion with the lowest percentage of fulfillment (25%) was the one limiting “protein dishes containing less than 70% of recommended weight for the portion of meat, fish or eggs” (3/20 max). It should be noted that dishes meeting this criterion are very diverse in terms of ingredients and therefore nutrients. For example, they include nuggets, lasagna, stuffed vegetables, quiches, and entirely vegetarian dishes such as tomato dumplings, cheese pizzas and Andalusian rice.

### 3.3. Nutritional Quality of the Observed Series

The observed series of 20 meals provided on average 14,232 kcal, or 712 kcal per meal, with energy from proteins, fats and carbohydrates equal to 18%, 33% and 46% respectively, and that from free sugars and SFAs equal to 4.5% and 12.5% respectively (results not shown).

The observed series provided 35.6% of recommended daily energy intake on average. The MAR, reached 49.3%, therefore clearly above the average threshold of 35.6% of recommended energy intake, indicating very good nutritional density in the observed series (Table 2).

**Figure A1:**
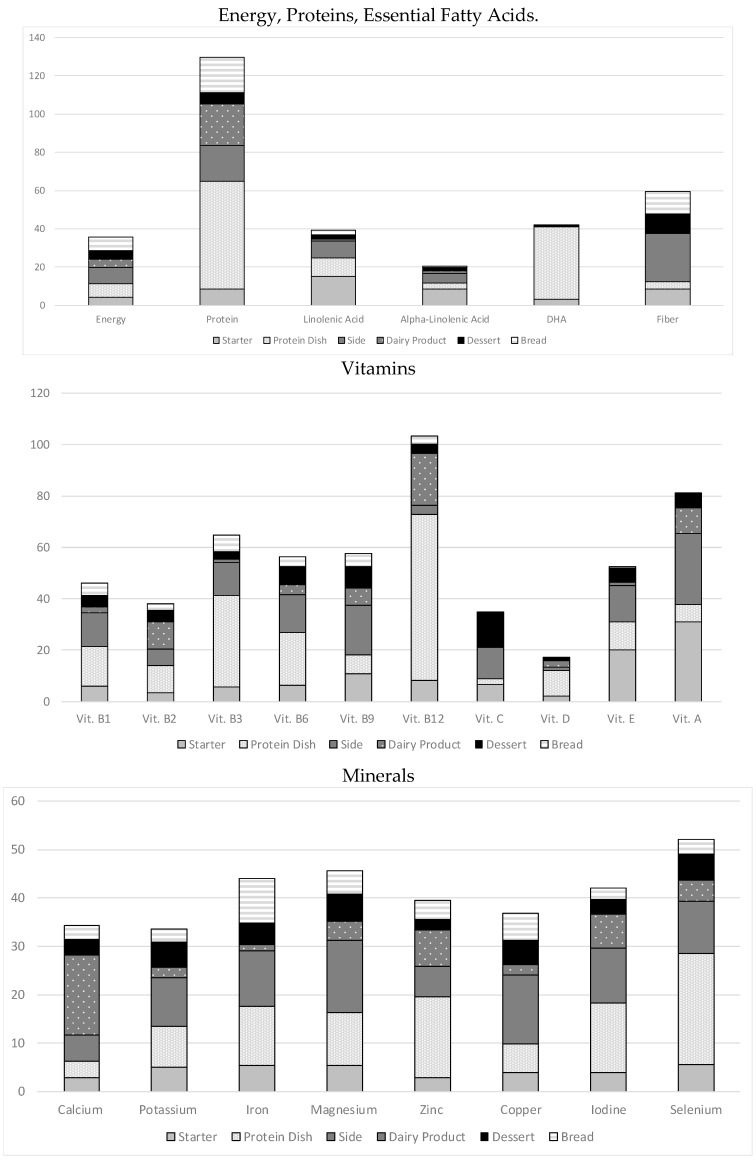
Average contributions of 5 meal components to the nutrient contents of the observed series (*n* = 40 series of 20 meals), expressed in % of daily recommended intakes (multiplied by 20).

When the nutrients to favor were examined separately, the observed series provided significantly more than the threshold of 35.6% of daily recommended intake for three-quarters of them. Vitamin C content (35.0%) was not significantly different from the 35.6% threshold, and calcium (34.2%) and potassium (33.6%) content were slightly but significantly below it. Only alpha-linolenic acid (20.4%) and vitamin D (17.3%) content were much lower than the 35.6% threshold. The five meal components contributed to coverage of recommended nutrient intakes differently depending on the nutrient considered (Figure A1). The starters were the main contributors of linoleic acid, alpha-linolenic acid, and vitamins A and E. The protein dishes provided the bulk of protein, DHA, and vitamins B3, B6, B12 and D, as well as iron, zinc, iodine and selenium. The side dishes were the main contributors of fiber, vitamin B9, potassium, magnesium and copper. Dairy products and desserts where the main contributors of calcium and vitamin C respectively.

### 3.4. Nutritional Quality of the Simulated Series

**Figure 3:**
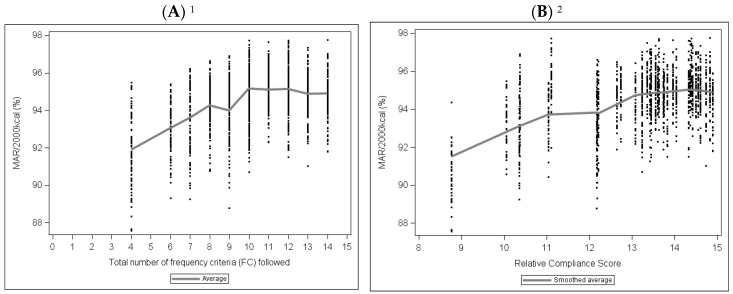
Mean Adequacy Ratio (MAR, expressed in % of adequacy for 2000 kcal) point clouds according to the level of compliance with frequency criteria (FC) estimated by the “Number of FC Followed” (**A**) and the “Relative Compliance Score” (**B**) in the “Compliance with Observed Series” series (*n* = 1600 series simulated by scenario 1, with recommended weights). ^1^ Spearman correlation: 0.36, *p* < 0.0001; ^2^ Spearman correlation: 0.34, *p* < 0.0001.

For the “Compliance with Observed Series” scenario, the level of compliance with FC, regardless of the score used, was positively correlated with MAR/2000 kcal (Figure 3).

This correlation reached a plateau—equal to 10 for the “Number of FC Followed” and 13 for the “Relative Compliance Score”—above which the correlation was no longer significant.

**Figure 4:**
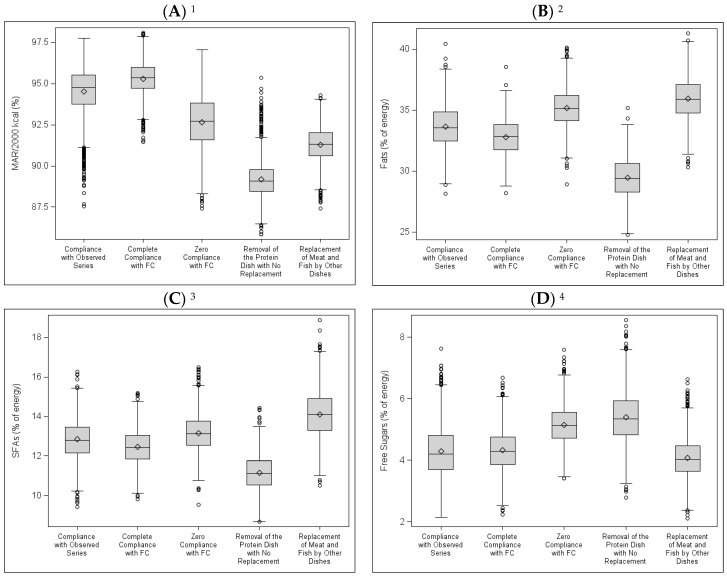
Nutritional quality of simulated series according to five scenarios: Box Plots (**A**) Mean Adequacy Ratio (MAR, % of adequacy for 2000 kcal); (**B**) Fats (% of energy); (**C**) Saturated Fatty Acids (SFAs, in % of energy); (**D**) Free Sugars (% of energy). ^1^ For MAR/2000 kcal, the averages are statistically different (*p* < 0.05) from one series to another. ^2^ For fats (% of energy), the averages are statistically different (*p* < 0.05) from one series to another, except between “Zero Compliance with FC” and “Replacement of Meat and Fish with Other Dishes”. ^3^ For SFAs (% of energy), the averages are statistically different from one series to another (*p* < 0.05). ^4^ For free sugars (% of energy), the averages are statistically different (*p* < 0.05) from one series to another except between “Complete Compliance with FC” and “Compliance with Observed Series”, and between “Complete Compliance with FC” and “Replacement of Meat and Fish with Other Dishes”.

Among the simulated series, the “Complete Compliance with FC” series had the highest MAR/2000 kcal on average (Figure 4).

In addition, in the “Complete Compliance with FC” series, the standard deviation of the MAR/2000 kcal was significantly lower than in the other series, suggesting that compliance with FC can prevent the occurrence of series with low MAR/2000 kcal values.

The “Zero Compliance with FC” series had lower nutritional quality (lower MAR/2000 kcal, plus high energy contribution from fats, SFAs and free sugars) than in the “Complete Compliance with FC” and “Compliance with Observed Series” scenario.

In the “Removal of the Protein Dish with No Replacement” series, the energy contribution from fats and SFAs was lower than in the other simulated series, the MAR/2000 kcal was significantly lower, and the energy contribution from free sugars was higher.

The “Replacement of Meat and Fish with Other Dishes” series were those in which the energy contribution from SFAs was highest, as was the contribution from fats, which was as high as in the “Zero Compliance with FC” series. The contribution from free sugars was not different from that of the “Complete Compliance with FC” series. The MAR/2000 kcal was higher than that of the “Removal of the Protein Dish with No Replacement” series, but it was lower than the MAR/2000 kcal of the other three simulated series, including the “Zero Compliance with FC” series.

## 4. Discussion

This study is the first one to clearly demonstrate the nutritional interest of the French school food guidelines. In addition, it shows that generalizing the service of meals without meat or fish would deteriorate the nutritional quality of school food service, advocating for a precise definition of the place of vegetarian dishes in school meals.

The observational part of the study was done on a sample of 40 series of 20 meals served in primary schools. The nutritional analysis of “observed” series revealed that they had good nutritional density because they provided proportionately more of the nutrients to favor (49.3% of recommendations) than calories (35.6% of daily recommendations). When nutrients were estimated separately, only alpha-linolenic acid and vitamin D were present in low quantities compared to recommendations. Yet, these nutrients are known for being largely deficient in French diets, for both children and adults [29], indicating that this is not a deficit specific to school food. In France, one school child out of three is deficient in vitamin D, advocating for systematic winter supplementation of this vitamin [32]. When it comes to alpha-linolenic acid, the GEM-RCN document recommends using mixtures of oils with high alpha-linolenic acid content [11]. In practice, this advice does not seem to be respected.

The analysis of the relationship between FC compliance level and nutritional quality required recourse to simulations. Indeed, it could not be done with the sample of observed series because of their relatively small number (*n* = 40) and because of the absence, in this sample, of series complying with all criteria and series not complying at all. The generation of a very large number of series close to what was observed (“Compliance with Observed Series”) showed that nutritional quality, estimated by the MAR/2000 kcal, increased with FC compliance level. The series simulated to comply with all 15 FC (“Complete Compliance with FC”) were nutritionally superior to all other series, notably the series close to what was observed (“Compliance with Observed Series”). In addition, the results show that complete compliance can prevent the occurrence of series of mediocre nutritional quality.

In the present study, observed compliance was partial, with only 9.7 out of the 15 frequency criteria followed on average in the sample of observed series. Yet, one can assume that, compared to all school food in France, compliance with frequency criteria was over-estimated in this sample because it was collected on a voluntary basis. This result seems to agree with studies done in other countries such as Australia [33], the United Kingdom [34] and other European countries [2], which have shown that school food guidelines, whether mandatory or not, are often difficult to implement and must be associated with measures aiming to facilitate their application. The aim is notably to help professionals correctly classify dishes in regard to existing recommendations or mandatory guidelines [2,35,36]. One can also assume that simplifying criteria could facilitate their application [2]. In this regard, our results suggest that it would be possible to revise the frequency criteria for school food in France so as to make them more flexible or reduce their number without losing effectiveness because there was no measurable nutritional benefit beyond compliance with 10 criteria (and a relative score equal to 13). However, more work would be needed to explore which kinds of simplification would be beneficial and acceptable.

One major advantage of the French school food guidelines is how they are formulated, which allows for the simultaneous consideration of several different characteristics of the dishes. Thus, some dishes are to be favored (minimal service frequency) while others are to be limited (maximal service frequency), but nothing is outlawed. In addition, the criteria apply to both the composition of dishes in certain ingredients and their nutrient content, and often combine two types of requirements (e.g., the “fish or fish-based preparations containing at least 70% fish and having a P/F ratio ≥ 2” criteria). School food guidelines are very different in the United Kingdom, where a sharp distinction is drawn between food-based standards (FBS) and nutrient-based standards (NBS) [8]. FBS (13 criteria) were first imposed in 2001, and then deemed insufficient and supplemented by NBS (14 criteria) [37,38]. However, even with these dual requirements—FBS and NBS—some nutritional goals, notably regarding iron, zinc and sodium, remained difficult to achieve [38]. Simplified standards (seven criteria on food groups to serve or limit) were introduced in 2014, because the previous ones were too difficult to understand and implement [34]. However, the new standards were in turn the subject of debate and criticism, notably regarding insufficient control of fat, saturated fatty acids, free sugars and sodium content [36]. Our study suggests that an approach such as the one adopted in French school food guidelines, which combine characteristics linked to the food, its nutrient composition and its service frequency in the same criteria, allow for good control of the nutritional quality of meals served to children.

Protein dishes are important contributors to greenhouse gas emissions and food waste in school meals [36], representing new challenges for this food sector [20]. However, the present study showed that their suppression would deteriorate the nutritional quality of series, as would replacing protein dishes containing meat or fish with dishes that do not contain meat or fish. The poor nutritional performance of the “Removal of the Protein Dish with No Replacement” scenario can be explained by the major contribution of protein dishes to the content of many essential nutrients (Figure A1). However, the poor performance of the “Replacement of Meat and Fish by Other Dishes” scenario is likely due to the mediocre nutritional quality of the dishes without meat or fish. Indeed, in the observed series, protein dishes without meat or fish were mostly based on eggs and/or cheese and grains, and lacked diversity (data not shown). Partners were therefore asked to provide additional technical files and recipes for protein dishes without meat or fish, especially plant-based protein dishes (see Appendix A), to be included in the simulation. However, despite this precaution, series modeled with the “no meat no fish” scenario were of lesser nutritional quality than with the observed scenario. This seems to confirm that it is nutritionally relevant to limit “protein dishes containing less than 70% of recommended weight for the portion of meat, fish or eggs” to 3/20 max. However, the large majority (75%) of the observed series did not fulfill this criterion, suggesting that it could be useful to modify it. The present results suggest that it could be interesting to include in the guidelines a positive definition of the expected nutritional characteristics of vegetarian dishes, and to determine how often they could be served to children. For instance, a new frequency criterion could be developed for dishes combining pulses and whole grains, considering that they are good sources of fibers, micronutrients and proteins, but also taking into account that they contain anti-nutritional factors that adversely influence the bioavailability of nutrients (i.e., the amount of ingested nutrient effectively available for the organism [39,40]). More generally, the present results show that meeting nutritional requirements is not necessarily compatible with reducing consumption of animal products, although the latter is recommended to lower the carbon footprint of our diet [36,41,42]. In a recent study, total replacement of animal products with products of plant origin improved some nutritional aspects (less sodium and SFAs, and more fiber) but decreased the content and/or the bioavailability of many essential nutrients (zinc, thiamine, vitamin A, vitamin B12, calcium and iron) [43].

The present study has its limitations. First, the data used in the descriptive portion were collected voluntarily and are therefore not representative of school food service in France. However, non-representativeness is not a major limitation because our goal was not to evaluate the nutritional quality of school meals in France but rather to assess the nutritional impact of complying with French school food guidelines and test some modifications in practices. Another limitation lies in the fact that the nutritional analysis was done on dishes as prepared in kitchens and served to children but not the portions actually eaten by children. We know, however, that offering healthier food at school encourages healthy eating among children [44,45]. For some dishes, the estimated nutrient content may have been inaccurate. Thus, for dishes with sauce, the quantity of sauce taken into account in the analysis was higher than the amount actually served to children because a non-negligible amount of the sauce remains in the serving dish. A final limit is that, in the scenarios of elimination of the protein dish or replacement of meat or fish dishes, it would be interesting to consider the bioavailability of nutrients when estimating the nutritional quality of the series. This consideration would likely have confirmed or even strengthened the observation of a nutritional worsening of the series in these scenarios because many key nutrients, such as iron, zinc and vitamin A, have greater bioavailability when they are of animal origin rather than plant origin [39,40], even though satisfactory bioavailability may be achieved in diets containing moderate quantities of animal products [46].

This study also presents strengths. First, precise data on meals and dishes actually served in schools were specifically collected for this study, making the database unique and rare. Indeed, it was produced based on real technical files and recipe cards—that is to say taking into account the nature and quantity of each ingredient in the dishes served by professionals—and not standard recipes or generic products systematically affiliated with a reading of dish names, as is habitually the case in other studies on school food [36,47,48]. With the exception of sodium and, to a lesser extent, sugars, whose quantities are often still difficult for professionals themselves to estimate, we can therefore consider that the estimated nutrient content was very precise. It was crucial to attain this level of precision to reliably estimate not only the nutritional quality of the series but also their level of compliance with frequency criteria because these criteria include the nutritional characteristics of dishes that depend directly on how the dishes are prepared. Another strength is the innovative and powerful approach that we elaborated to test the nutritional impact of the French school food guidelines. This approach allowed for realistic testing of the simultaneous compliance with the 15 frequency criteria, which had until now only been explored separately and partially, notably because of the lack of series complying fully with them [47].

## 5. Conclusions

In conclusion, this study revealed the good nutritional quality of a sample of series of meals served at primary schools in France and the nutritional relevance of French school food guidelines. The results suggest that there may be room for intelligently increasing the flexibility of these guidelines because no nutritional benefit was observed above a relative score of 13 (out of 15 frequency criteria). However, in the event of a wide gap from frequency criteria guidelines, whether for all criteria or those addressing protein dish service, a risk of worsening nutritional quality was shown. Thus, in the absence of a precise definition of the nutritional characteristics of protein dishes without meat or fish, the generalization of their service in schools could harm the nutritional balance of meals served to children.

## Figures and Tables

**Table 1:** The 15 frequency criteria for school meal service in France [10,13] and the percentage of series fulfilling each criterion among the 40 observed series of 20 meals.

Criteria	Component(s) Concerned	Expected Frequency	% of Observed Series Fulfilling the Criterion ^1^
Starters containing more than 15% fat	starter	4/20 max	82.5%
Raw vegetable or fruit dishes containing at least 50% vegetables or fruits	starter, side dish	10/20 min	70%
Dishes to fry or pre-fried dishes containing more than 15% fat	protein dish, side dish	4/20 max	100%
Protein dishes with a ratio of P/F ^1^ ≤ 1	protein dish	2/20 max	55%
Fish or fish-based dishes containing at least 70% fish and having a ratio of P/F ≥ 2	protein dish	4/20 min	60%
Unground beef, veal or lamb, and offal	protein dish	4/20 min	77.5%
Preparations or ready-to-eat dishes containing less than 70% of the recommended weight for the portion of meat, fish or eggs	protein dish	3/20 max	25%
Vegetables, other than pulses, alone or in a mixture containing at least 50% vegetables	side dish	=10/20	27.5%
Pulses, starches or grains, alone or in a mixture containing at least 50% pulses, starches or grains	side dish	=10/20	27.5%
Cheese containing at least 150 mg of calcium per portion	starter, dairy product	8/20 min	77.5%
Cheese with a calcium content of more than 100 mg and less than 150 mg per portion	starter, dairy product	4/20 min	57.5%
Dairy (fresh dairy products, dairy-based desserts) containing more than 100 mg of milk calcium and less than 5 g of fat per portion	dairy product, dessert	6/20 min	40%
Desserts containing more than 15% fat	dessert	3/20 max	95%
Desserts or dairy products containing more than 20 g of total simple sugars per portion and less than 15% fat	dairy product, dessert	4/20 max	100%
Raw fruit dessert 100% raw fruit without added sugars	dessert	8/20 min	77.5%

^1^ Ratio P/F = protein (g/100 g)/fat (g/100 g).

**Table 2:** Recommended daily intakes of energy and nutrients (proteins, essential fatty acids (FAs), fiber, vitamins and minerals) included in the calculation of the Mean Adequacy Ratio (MAR) and percentages of these recommendations in the observed series of 20 meals (*N* = 40), expressed per meal ^a^.

	Recommended Daily Intake	Average Content (% of Recommendation, Expressed Per Meal)	Standard Deviation
Energy	1996 kcal [24,28,29]	35.6	2.7
Protein	25 g [28,29]	129.8	8.5
Linoleic Acid	8.9 g [30]	39.3	10.2
α-Linolenic Acid	2.2 g [30]	20.4	8.3
DHA	152 mg [30]	42.2	18.0
Fiber	13 g [24]	59.5	6.2
Vitamin B1	0.8 mg [24]	46.3	5.2
Vitamin B2	1.2 mg [24]	38.2	3.4
Vitamin B3	9 mg [24]	64.9	8.9
Vitamin B6	1 mg [24]	56.4	6.0
Vitamin B9	201 µg [24]	57.6	6.7
Vitamin B12	1.4 µg [24]	103.4	15.4
Vitamin C	89 mg [24]	35.0	6.3
Vitamin D	5 µg [24]	17.3	4.1
Vitamin E	9.1 mg [24]	52.5	15.7
Vitamin A	501 µg [24]	81.0	14.2
Calcium	924 mg [24]	34.2	4.1
Potassium	2892 mg [31]	33.6	3.8
Iron	8.2 mg [24]	44.0	4.3
Magnesium	203 mg [24]	45.6	3.8
Zinc	9.2 mg [24]	39.5	4.1
Copper	1.2 mg [24]	36.8	3.3
Iodine	120 µg [24]	42.1	6.7
Selenium	39 µg [24]	52.0	6.1
MAR (%) ^b^		49.3	3.7

^a^ Here, “meal” is defined as the average value for a complete series of 20 meals divided by 20. ^b^ Estimated according to equation 1.

## Appendix A. Detailed Description of the Five Scenarios

In the first scenario—“Compliance with Observed Series”—series of meals were generated to produce series that were close to what was observed while gaining high statistical strength. Each of the 40 observed series was repeated 40 times by keeping the same service frequency of the meals while including different dishes. For example, if an observed series contained five “starters containing more than 15% fat”, all the 40 series simulated based on this series also contained five “starters containing more than 15% fat” but each dish introduced in each simulated series was selected using a weighted random sampling (for example, if a grated celery salad containing more than 15% fat was served ten times more often than a cheese puff pastry containing more than 15% fat in the observed series, then grated celery salad was 10 times more likely to be selected than cheese puff pastry in simulated series). In all, 1600 (40 × 40) series were simulated in this scenario.

In the second scenario—“Complete Compliance with FC”—series of meals in which all 15 FC were followed exactly were generated. All of the structures compatible with compliance with FC while remaining within realistic frequency ranges were first counted. These realistically complying ranges were identified from 40 observed series as follows: For example, if all the observed series respecting the frequency criterion “starters containing more than 15% fat: 4/20 max” always contained at least two (and never 0 or 1) “starters with more than 15% fat”, then the frequency range realistically complying with the criterion would be 2–4 (even though the authorized range was 0–4). In all, 3,024,000 structures of series realistically complying with all 15 FC were enumerated, within which 1600 structures were derived so as to obtain the same representativeness of compliance with each criterion (e.g., if, among the observed series complying with the criterion “starters containing more than 15% fat: 4/20 max”, we observed 25% of them contained two starters containing more than 15% fat, 25% containing 3 starters containing more than 15% fat, and 50% containing 4 starters containing more than 15% fat, then these proportions were maintained in the simulation). Then, as previously, dishes were allocated to each of the 1600 structures, taking into account the classification of each dish in regard to the 15 FC and its degree of appearance in the observed series.

In the third scenario—“Zero Compliance with FC”—series that complied with none of the regulatory frequency criteria while remaining within realistic and representative frequency ranges were generated. For example, if no observed series contained 10 “starters containing more than 15% fat”, then this was also the case for the “Zero Compliance with FC” series. In addition, if, among the observed series that did not comply with the “starter containing more than 15% fat: 4/20 max”, 75% had five and 25% had six, then these proportions were repeated in the simulation. This concern for realism also led us to decide that if a frequency criterion was followed in all observed series, it would also be followed in the “Zero Compliance with FC” series.

In the fourth scenario—“Removal of the Protein Dish with No Replacement”—series of meals were generated by removing all protein dishes from the “Complete Compliance with FC” series (generated in the second scenario).

In the fifth and last scenario—“Replacement of Meat and Fish by Other Dishes”—series of meals were generated by randomly replacing the protein dishes and starters containing meat or fish in the “Complete Compliance with FC” series (generated in the second scenario) with protein dishes and starters that did not contain meat or fish. It should be noted that dishes “without meat or fish” are defined as “vegetarian” under the GEM-RCN’s Nutrition Recommendations [13]. In order to have sufficient diversity in the simulated series for this scenario, 32 dishes served as protein dishes without meat or fish and not included in the dishes present in the 40 series of 20 meals were specifically collected from the partners. These additional technical files corresponded to dishes actually served in schools as alternatives to meat or fish dishes.

## Appendix B

**Table A1:** Categorization and distribution of dishes per meal component in the 40 observed series.

Component	Category	Number
Starter	Vegetables	521
	Starches	160
	Deli Meats	37
	Cheese	29
	Fruits	23
	Eggs	10
	Fish	9
Protein Dish	Pork, Poultry	305
	Beef, Veal, Lamb	243
	Fish	211
	Eggs	34
	Starches	6
	Vegetables	1
Side Dish	Starches	441
	Vegetables	357
Dairy Product	Cheese	556
	Dairy Products	220
	Dairy Desserts	5
	Sweetened Foods	1
Dessert	Fruits	478
	Sweetened Foods	164
	Dairy Desserts	111
	Dairy Products	17
	Starches	16

## Appendix C

**Table A2:** Example of calculation of MAR and MAR/2000 kcal for one series containing 14,871 kcal.

		MAR Estimation			MAR/2000 kcal Estimation			
	Nutritional Content of the Series	Recom Mended 20 Days Intake	Ratio	Ratio > 100 Replaced with 100	Content for 2000 kcal	Recom Mended Daily Intake	Ratio	Ratio > 100 Replaced with 100
Protein (g)	683.66	500	136.73	100.00	91.94	25	367.78	100
Linoleic Acid (g)	85.76	178	48.18	48.18	11.53	8.9	129.59	100
α-Linolenic Acid (g)	15.53	44	35.29	35.29	2.09	2.2	94.91	94.91
DHA (mg)	1973.28	3040	64.91	64.91	265.39	152	174.60	100
Fiber (g)	188.41	260	72.46	72.46	25.34	13	194.91	100
Vitamin B1 (mg)	7.20	16	44.99	44.99	0.97	0.8	121.01	100
Vitamin B2 (mg)	9.86	24	41.08	41.08	1.33	1.2	110.49	100
Vitamin B3 (mg)	118.20	180	65.67	65.67	15.90	9	176.63	100
Vitamin B6 (mg)	11.18	20	55.92	55.92	1.50	1	150.40	100
Vitamin B9 (µg)	2464.13	4020	61.30	61.30	331.40	201	164.88	100
Vitamin B12 (µg)	24.11	28	86.12	86.12	3.24	1.4	231.64	100
Vitamin C (mg)	616.79	1780	34.65	34.65	82.95	89	93.20	93.20
Vitamin D (µg)	19.79	100	19.79	19.79	2.66	5	53.23	53.23
Vitamin E (mg)	128.37	182	70.53	70.53	17.26	9.1	189.72	100
Vitamin A (µg)	6942.86	10,020	69.29	69.29	933.74	501	186.38	100
Calcium (mg)	6067.86	18,480	32.83	32.83	816.06	924	88.32	88.32
Potassium (mg)	19,425.11	57,840	33.58	33.58	2612.48	2892	90.33	90.33
Iron (mg)	73.00	164	44.51	44.51	9.82	8.2	119.73	100
Magnesium (mg)	1944.72	4060	47.90	47.90	261.54	203	128.84	100
Zinc (mg)	73.85	184	40.14	40.14	9.93	9.2	107.96	100
Copper (mg)	9.93	24	41.39	41.39	1.34	1.2	111.34	100
Iodine (µg)	956.99	2400	39.87	39.87	128.71	120	107.25	100
Selenium (µg)	516.99	780	66.28	66.28	69.53	39	178.28	100
			MAR=	52.90		MAR/2000 kcal=		96.52

## References

[1] European Union (EU) EU Action Plan on Childhood Obesity 2014–2020. https://ec.europa.eu/health/sites/health/files/nutrition_physical_activity/docs/childhoodobesity_actionplan_2014_2020_en.pdf.

[2] Caldeira S., Storcksdieck Genannt Bonsmann S., Bakogianni I., Gauci C., Calleja A., Furtado A. (2017). Public Procurement of Food for Health: Technical Report on the School Setting.

[3] Storcksdieck S., Kardakis T., Wollgas J., Nelson M., Caldeira S. (2014). Mapping of National School Food Policies across the EU28 plus Norway and Switzerland.

[4] WHO Regional Office for Europe (2006). Food and Nutrition Policy for Schools: A Tool for the Development of School Nutrition Programmes in the European Region.

[5] Commission de L’environnement de La Santé Publique et de La Sécurité Alimentaire Rapport Sur le Livre Blanc Sur La Nutrition, La Surcharge Pondérale et L’obésité (2007/2285(INI)). http://www.europarl.europa.eu/sides/getDoc.do?pubRef=-//EP//NONSGML+REPORT+A6-2008-0256+0+DOC+PDF+V0//FR.

[6] Lafay L., Volatier J.-L., Martin A. (2002). La restauration scolaire dans l’enquête INCA, 2e partie: Les repas servis en restauration scolaire: Apports nutritionnels, alimentaires et impact sur la nutrition des enfants. Cah. Nutr. Diet..

[7] Evans C.E., Mandl V., Christian M.S., Cade J.E. (2016). Impact of school lunch type on nutritional quality of English children’s diets. Public Health Nutr..

[8] Weichselbaum E., Gibson-Moore H., Ballam R., Buttriss J.L. (2011). Nutrition in schools across Europe: A summary report of a meeting of European Nutrition Foundations, Madrid, April 2010. Nutr. Bull..

[9] République Française Décret du 30 Septembre 2011–1227 Relatif à la Qualité Nutritionnelle des Repas Servis Dans le Cadre de la restauration scolaire. https://www.legifrance.gouv.fr/eli/decret/2011/9/30/2011-1227/jo/texte.

[10] République Française Arrêté du 30 Septembre 2011 Relatif à la Qualité Nutritionnelle Des Repas Servis Dans le Cadre de la Restauration Scolaire. https://www.legifrance.gouv.fr/eli/arrete/2011/9/30/AGRG1032380A/jo/texte.

[11] Ministère de l’Economie (2007). Groupe d’Etude Marchés Restauration collective et Nutrition (GEM-RCN); Recommandation Relative à La Nutrition du 4 Mai 2007.

[12] Ministère de l’Economie, Groupe d’Etude des Marchés de Restauration Collective et de Nutrition (GEM-RCN) (2011). Recommandation Nutrition Complétée et Mise à Jour au 10 Octobre 2011.

[13] Ministère de l’Economie, Direction des Affaires Juridiques, Observatoire Economique de l’Achat Public, Groupe d’Etude des Marchés Restauration Collective et de Nutrition (GEM-RCN) (2015). Recommandation Nutrition, Version 2.0 Juillet 2015.

[14] Dubuisson C., Lioret S., Calamassi-Tran G., Volatier J.L., Lafay L. (2009). School meals in French secondary state schools with regard to the national recommendations. Br. J Nutr..

[15] Bertin M., Lafay L., Calamassi-Tran G., Volatier J.L., Dubuisson C. (2011). Schools meals in French secondary state schools: compliance to national recommendations and schools catering patterns. Rev. Epidemiol. Sante Publique.

[16] Food and Agriculture Organization of the United Nations (FAO) (2017). Nutrition-Sensitive Agriculture and Food Systems in Practice Options for Intervention.

[17] Maillot M., Darmon N., Darmon M., Lafay L., Drewnowski A. (2007). Nutrient-Dense Food Groups Have High Energy Costs: An Econometric Approach to Nutrient Profiling. J. Nutr..

[18] Masset G., Soler L.G., Vieux F., Darmon N. (2014). Identifying sustainable foods: the relationship between environmental impact, nutritional quality, and prices of foods representative of the French diet. J. Acad. Nutr. Diet..

[19] Tilman D., Clark M. (2014). Global diets link environmental sustainability and human health. Nature.

[20] Agence de L’environnement et La Maîtrise de L’énergie (ADEME) (2016). Approche du Coût Complet des Pertes et Gaspillage Alimentaire en Restauration Collective.

[21] Derbyshire E.J. (2016). Flexitarian Diets and Health: A Review of the Evidence-Based Literature. Front. Nutr..

[22] ANSES French Food Composition Table Ciqual 2013. https://ciqual.anses.fr/#.

[23] Guthrie H.A., Scheer J.C. (1981). Validity of a dietary score for assessing nutrient adequacy. J. Am. Diet. Assoc..

[24] Martin A. (2001). Apports Nutritionnels Conseillés Pour La Population Française.

[25] Ministère de L’éducation Nationale De l’enseignement Supérieur et de La Recherche (2014). Repères et Références Statistiques Sur Les Enseignements, La Formation et la Recherche.

[26] World Health Organization (WHO) (2003). Diet, Nutrition and the Prevention of Excess Weight Gain and Obesity.

[27] Drewnowski A. (2005). Concept of a nutritious food: toward a nutrient density score. Am. J. Clin. Nutr..

[28] Potier de Courcy G., Frelut M.L., Fricker J., Martin A., Dupin H. (2003). Besoins nutritionnels et apports conseillés pour la satisfaction de ces besoins. Encycl. Med. Chir..

[29] Lafay L. (2007). Etude Individuelle et Nationale sur les Consommations Alimentaires, INCA 2 (2006–2007). https://www.anses.fr/fr/system/files/PASER-Ra-INCA2.pdf.

[30] ANSES (2011). Actualisation des Apports Nutritionnels Conseillés Pour les Acides Gras-Rapport D’expertise Collective.

[31] ANSES (2015). Evaluation des Apports en Vitamines et Minéraux Issus de L’alimentation Non Enrichie, de L’alimentation Enrichie et des Compléments Alimentaires Dans la Population Française: Estimation des Apports Usuels, des Prévalences D’inadéquation et des Risques.

[32] Mallet E., Gaudelus J., Reinert P., Stagnara J., Benichou J., Basuyau J.P., Maurin M., Cordero J., Roden A., Uhlrich J. (2014). Vitamin D status in 6- to 10-year-old children: A French multicenter study in 326 children. Arch. Pediatr..

[33] Ardzejewska K., Tadros R., Baxter D. (2012). A descriptive study on the barriers and facilitators to implementation of the NSW (Australia) Healthy School Canteen Strategy. Health Educ. J..

[34] Dimbleby H., Vincent J. (2013). The School Food Plan. http://www.schoolfoodplan.com/wp-content/uploads/2013/07/School_Food_Plan_2013pdf.

[35] Yoong S.L., Nathan N., Wolfenden L., Wiggers J., Reilly K., Oldmeadow C., Wyse R., Sutherland R., Delaney T., Butler P. (2016). CAFE: A multicomponent audit and feedback intervention to improve implementation of healthy food policy in primary school canteens: A randomised controlled trial. Int. J. Behav. Nutr. Phys. Act..

[36] Wickramasinghe K.K., Rayner M., Goldacre M., Townsend N., Scarborough P. (2016). Contribution of healthy and unhealthy primary school meals to greenhouse gas emissions in England: Linking nutritional data and greenhouse gas emission data of diets. Eur. J. Clin. Nutr..

[37] Haroun D., Wood L., Harper C., Nelson M. (2011). Nutrient-based standards for school lunches complement food-based standards and improve pupils’ nutrient intake profile. Br. J. Nutr..

[38] Haroun D., Harper C., Wood L., Nelson M. (2011). The impact of the food-based and nutrient-based standards on lunchtime food and drink provision and consumption in primary schools in England. Public Health Nutr..

[39] Gibson R.S. (2007). The role of diet- and host-related factors in nutrient bioavailability and thus in nutrient-based dietary requirement estimates. Food Nutr. Bull..

[40] Millward D.J., Garnett T. (2010). Plenary Lecture 3: Food and the planet: nutritional dilemmas of greenhouse gas emission reductions through reduced intakes of meat and dairy foods. Proc. Nutr. Soc..

[41] Vieux F., Darmon N., Touazi D., Soler L.G. (2012). Greenhouse gas emissions of self-selected individual diets in France: Changing the diet structure or consuming less?. Ecol. Econ..

[42] Payne C.L., Scarborough P., Cobiac L. (2016). Do low-carbon-emission diets lead to higher nutritional quality and positive health outcomes? A systematic review of the literature. Public Health Nutr..

[43] Seves S.M., Verkaik-Kloosterman J., Biesbroek S., Temme E.H. (2017). Are more environmentally sustainable diets with less meat and dairy nutritionally adequate?. Public Health Nutr..

[44] Niebylski M.L., Lu T., Campbell N.R., Arcand J., Schermel A., Hua D., Yeates K.E., Tobe S.W., Twohig P.A., L’Abbe M.R. (2014). Healthy food procurement policies and their impact. Int. J. Environ. Res. Public Health.

[45] Wang D., Stewart D. (2013). The implementation and effectiveness of school-based nutrition promotion programmes using a health-promoting schools approach: A systematic review. Public Health Nutr..

[46] Perignon M., Barre T., Gazan R., Amiot M.J., Darmon N. (2018). The bioavailability of iron, zinc, protein and vitamin A is highly variable in French individual diets: Impact on nutrient inadequacy assessment and relation with the animal-to-plant ratio of diets. Food Chem..

[47] Bertin M., Lafay L., Calamassi-Tran G., Volatier J.L., Dubuisson C. (2012). School meals in French secondary state schools: do national recommendations lead to healthier nutrition on offer?. Br. J. Nutr..

[48] Nicholas J., Wood L., Harper C., Nelson M. (2013). The impact of the food-based and nutrient-based standards on lunchtime food and drink provision and consumption in secondary schools in England. Public Health Nutr..

